# A tracked robot with novel bio-inspired passive “legs”

**DOI:** 10.1186/s40638-017-0070-6

**Published:** 2017-11-13

**Authors:** Bo Sun, Xingjian Jing

**Affiliations:** 1 Department of Mechanical Engineering, Hong Kong Polytechnic University, Hong Kong, China; 2 Hong Kong Polytechnic University Shenzhen Research Institute, Shenzhen, China

**Keywords:** Track-based robots, Mobile robots, Bio-inspired X-shaped structures, Stability control

## Abstract

For track-based robots, an important aspect is the suppression design, which determines the trafficability and comfort of the whole system. The trafficability limits the robot’s working capability, and the riding comfort limits the robot’s working effectiveness, especially with some sensitive instruments mounted on or operated. To these aims, a track-based robot equipped with a novel passive bio-inspired suspension is designed and studied systematically in this paper. Animal or insects have very special leg or limb structures which are good for motion control and adaptable to different environments. Inspired by this, a new track-based robot is designed with novel “legs” for connecting the loading wheels to the robot body. Each leg is designed with passive structures and can achieve very high loading capacity but low dynamic stiffness such that the robot can move on rough ground similar to a multi-leg animal or insect. Therefore, the trafficability and riding comfort can be significantly improved without losing loading capacity. The new track-based robot can be well applied to various engineering tasks for providing a stable moving platform of high mobility, better trafficability and excellent loading capacity.

## Introduction

In the suspension design of track-based robots, including engineering machinery, most methods are motivated by tank suspension design. The tank suspension design is rather complicated, which has shaped various tracked vehicle designs, including tanks and other vehicles, conception of which came out in WW1. The idea of a suspension system to dampen impacts on the chassis and to decrease the force on the tracks from terrain has been well known, with many approaches leading to many effects. Several classic tank suspension designs are briefly discussed as follows.

Initially, the first tank was designed without suspension used in WW1 on the British tanks. Later variations had the tracks on big modules connected to the hull with a girder, like the Whippet [1]. From the period of WW1 until WW2, the direct coil bogie [2] was adopted which is a form of suspension, with single or multiple coil springs carrying a bogie. This was a big improvement, but still only capable of very minimal travel. Later versions were invented to allow it to pivot and help with interconnection, but still limited by the size of the compressed spring [2].

Later on, leaf-sprung bogies are introduced in the tank suspension design [3, 4]. The system is made of a pair of wheels carried on leaf sprigs or leaf-sprung arms. When one wheel is raised like by contacting terrain, the other is lowered to keep uniform track pressure, which is rather light, maintenance-friendly, has decent travel length and keeps the tracks from slipping. Since the friction of the leaves sliding over each other dampens oscillation, no shock absorbers are needed. This cheap and effective system was quickly adopted widely and modified into various forms [3, 4].

During the war period, French developed a standardized system of rubber springs used in their tanks, which consist of a pair of wheels springing against each other [5]. Rubber has a natural dampening property and is not prone to breaking like steel. But the ability to stand fire and shell fragment is limited. Similar systems are used today in vocational trucks and bulldozers. The volute spring was a very durable system consisted of suspension arms and coiled strips of metal, which was developed and used only on US tanks [6]. As the metal layers glided over each other, it was a strong system and the leverage of bogie arms provided a decent travel distance. Later variants used the horizontal volute springs, springing one wheels off another to interconnect, which contributed to surprisingly long track life combined with the advanced and well-insulated American running gear design.

The Horstman bogie is almost like the US suspension introduced above, but use a coil spring instead of a volute spring. This system is very comparable to torsion bars, being a tiny bit stiffer but also having better interconnection. This system was retained on the Chieftain tank [7] and several other vehicles. The Christie system used a bell crank to transfer arm rotation to spring to allow that longer springs can be used, which was invented in American in 1930s. But the Soviets firstly used this system on the BT and T-34 [8]. This system can provide high ride quality due to its long spring travel length. Original Christie tanks also had a chain drive option, so they could run without their tracks on roads. It also has the advantages in maintenance and protection of the chassis.

The Bell Crank system is the one with a pair of wheels on a bell crank, each bogie connected by a short arm and lever to a central spring running the tank length [9]. This system had too much interconnection but decent travel length and tended to make the tank sway back and forth a lot in motion. The single and double Torsion Bar system is a common modern-type tank suspension and can be found today on many fast track layers. The original structure and concept appeared on the German tiger tanks [10]. A steel bar runs under the bottom of the tank and twists with a short trailing arm connected to it and carrying a wheel. As the wheels hit bumps, the bar twists to absorb the impact. This system is compact, light, simple to manufacture and can provide decent travel length.

The hydropneumatic system adopts flexible and soft gas as a springing medium in cylinders for each wheel to damp and absorb the shock [11]. The system is actively controlled by the driver to achieve depression and elevation and adjusted by the computer to make the chassis steady while traveling. This is a very complex but effective and generally light system that promotes track life, crew comfort, and low mechanical damage due to terrain.

**Fig. 1:**
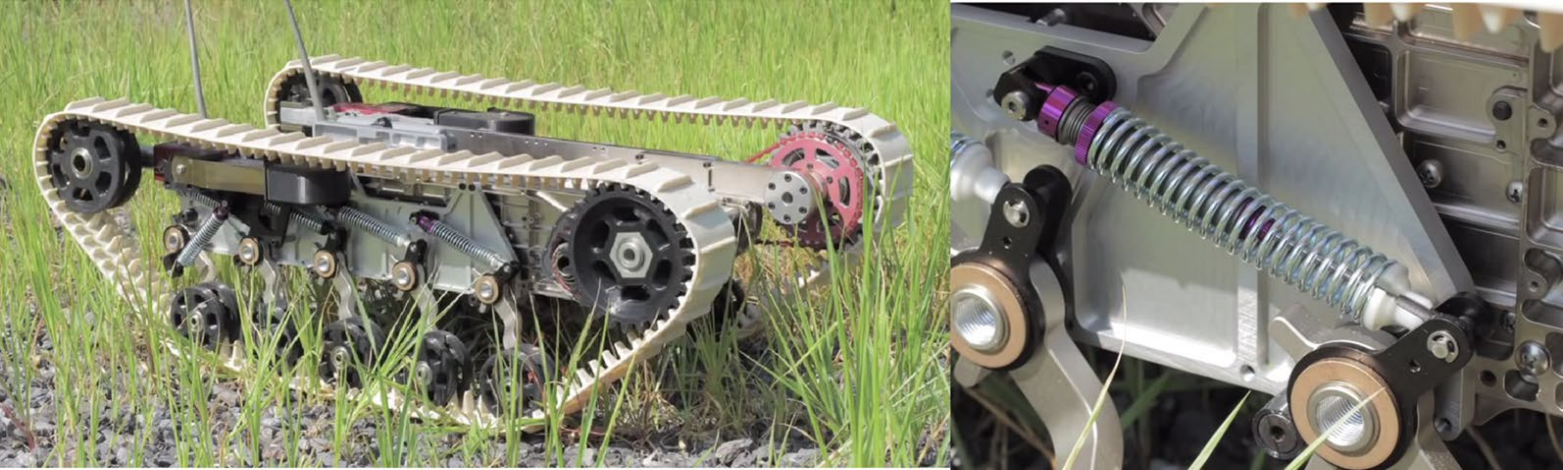
DARPA track-based robot suspension design [20]

Nowadays, most modern track-based tank designs aim at high trafficability to achieve stair climbing or all-terrain mobility. Few suspensions are designed for a more stable platform during traveling. A benchmark example is the one developed by DARPA (Fig. 1) which considered ground vibration and adopted a new arrangement of suspension design. However, compared to the Christie suspension, the DARPA design can be regarded as a further development or optimization and the robot is good only for heading to one direction.

For mobile robot design, compactness, lightweight, mobility, stability, loading capacity, and so on are all needed to be considered. Especially for those intelligent moving platforms, they are usually expected to carry sensitive equipment for different tasks, requiring more riding comfort and stability, etc. Even though many robots adapted the active vibration isolation approach, its drawbacks of large size, high energy consumption, expensive manufacturing costs, and so on limited the development and application.

Therefore, novel suspension systems of high performance but in passive control manner are highly relevant to the related areas. To this aim, a novel passive bio-inspired limb-like suspension system is developed in this study, which is completely controlled in a purely passive manner, but can achieve high loading capacity, high stability, high trafficability on tough ground, similar to a multi-leg insects or crabs.

The rest of the paper is organized as follows: “The novel bio-inspired passive suspension structure” and “Structural modeling and suspension analysis” sections introduce the bio-inspired suspension design and its mathematical modeling. “ADAMS model” section presents some simulation results of the novel suspension structure. Experiment results are introduced in “Experiment study” section. A conclusion is drawn thereafter.

## The novel bio-inspired passive suspension structure

**Fig. 2:**
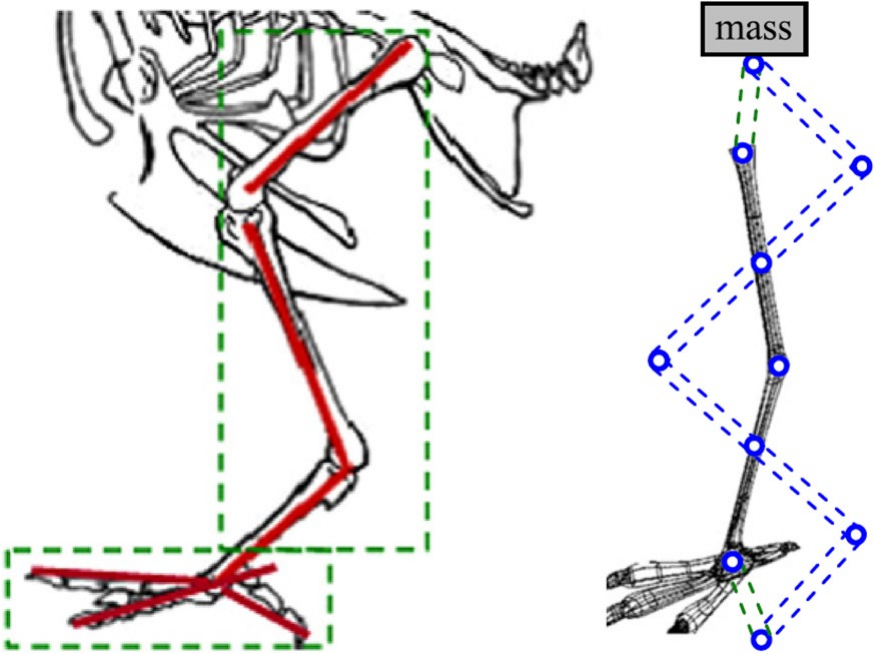
Bird’s limb structure and the bio-inspired X-shaped structure

Plenty structures and materials in the nature provide fantastic solutions in vibration isolation and control. It is noticed that legs or limbs of animals are very easy to suppress vibration and mitigate shock impact during moving or jumping due to the very special leg or limb structure. Inspired by the bird’s leg structure, a new bio-inspired limb-like or X-shaped anti-vibration structure (Fig. 2) was proposed and studied recently in [12–17].

The X-shaped anti-vibration structure turned out to be very effective to isolate shock and vibration and keep the upper platform steady. It has been shown that the X-shaped structure was proved to be a quasi-zero-stiffness (QZS) system, which had the characteristic that can isolate a much broader frequency band of vibration range than traditional linear isolation systems [12–17]. The QZS system can be optimized to keep the static stiffness high and dynamic stiffness low by properly designing the system parameters, which means the system can remain a high loading capacity while most of vibrations can be isolated during motions. These high static stiffness and low dynamic stiffness properties are exactly needed in the design of the novel robot suspension.

**Fig. 3:**
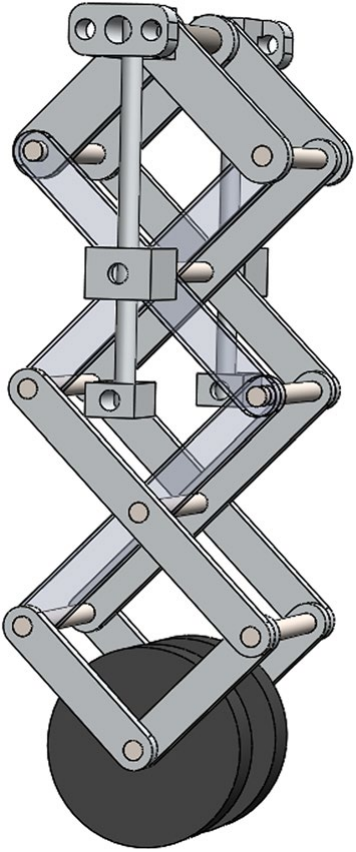
3-Layer X-structured suspension

By applying the bio-inspired X-shaped structure to the suspension design in the track-based robot, each loading wheel will be connected to a specially designed X-shaped structure and then fixed to the robot body. The single-wheel suspension is shown in Fig. 3, which is constructed by aluminum rods in a X-shaped form with springs installed horizontally, in which a central rod provides the vertical constraint to make the loading wheel only move vertically from the ground up to 10 cm in height (from the working position).

**Fig. 4:**
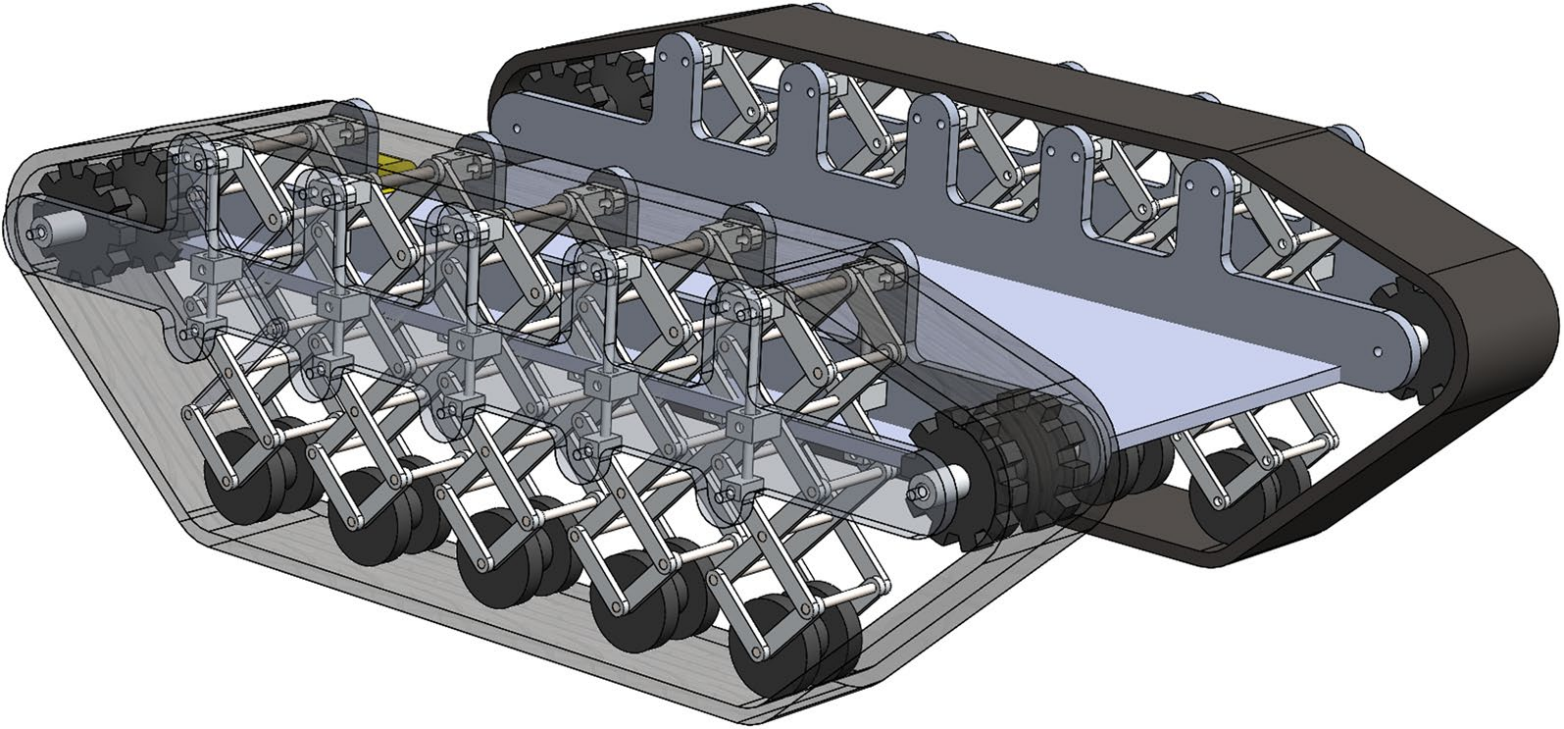
Overall 3-layer bio-inspired leg track-based robot

By combining all independent bio-inspired X-structured suspensions together, the whole suspension of the robot is shown in Fig. 4. Benefited by the single X-structured suspension, the whole robot can have a decent load capacity and vibration isolation performance to absorb the disturbance on road. Also, the long travel distance of the suspension can ensure the robot easily to overcome a rather large obstacle on road without losing the stability of the upper platform.

**Fig. 5:**
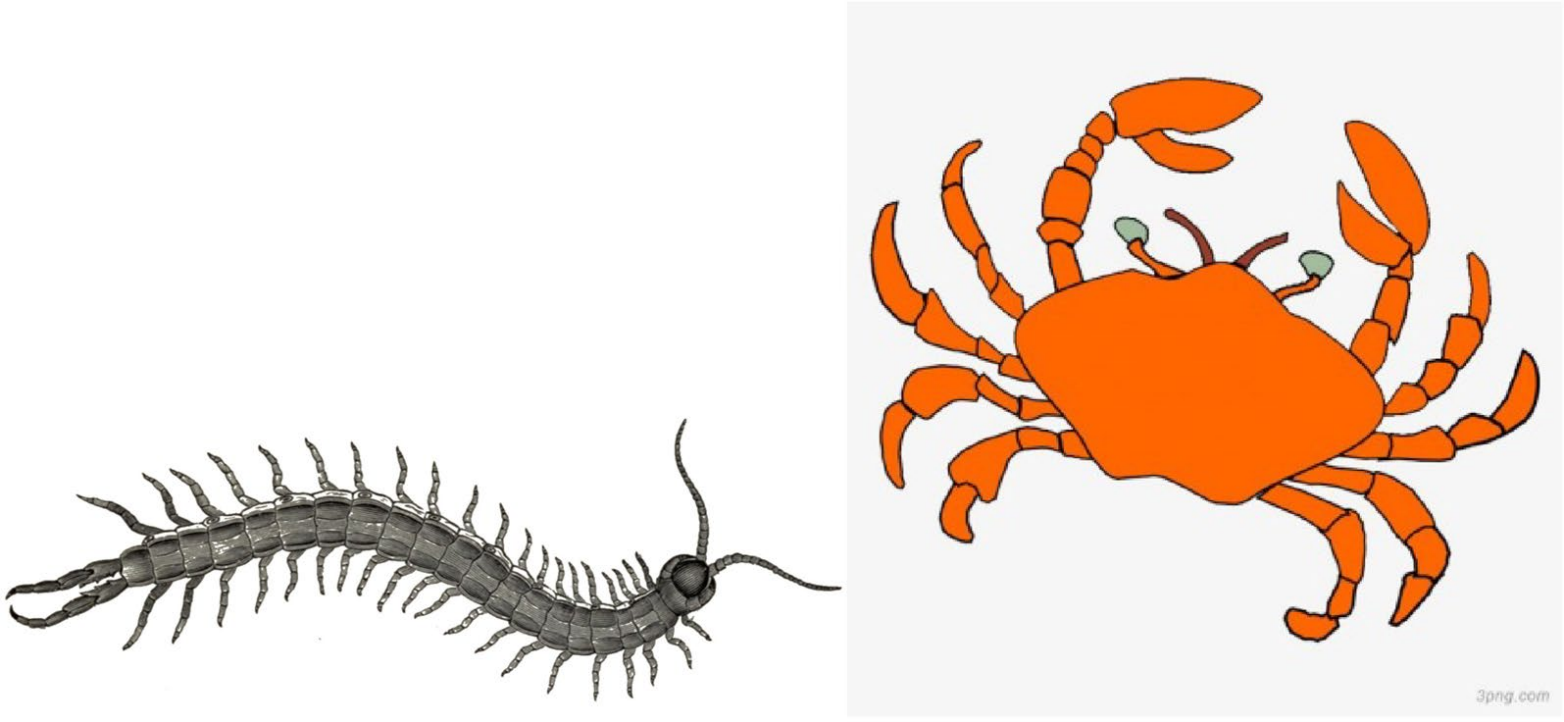
Multi-leg animals can handle their stability more easily

This would be similar to many multi-leg insects or animals (Fig. 5). Each leg may not be strong enough to support the body weight, but all legs together are redundant. Although one leg is stepped on an obstacle, the other legs can still maintain the stability of the body. Importantly, the new suspension design of the robot in Fig. 4 is totally in a passive control manner. The next section will provide more understanding about the stiffness property of the single X-structured suspension and explain more about why such a passive suspension can simultaneously achieve high loading capacity but low dynamic stiffness and therefore lead to an excellent passive vehicle suspension.

## Structural modeling and suspension analysis

### Static stiffness analysis

**Fig. 6:**
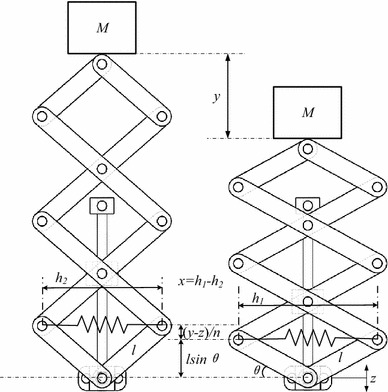
3-Layer bio-inspired leg suspension model

Referring to Fig. 6 for all structural parameter definitions, and from Fig. 6 the geometry relationship [12–17] can be obtained as

$$\tan \left( {\theta + \varphi } \right) = \frac{2l\sin \theta + y/3}{2l\cos \theta - x}$$

$$\left( {2l} \right)^{2} = \left( {2l\sin + y/3} \right)^{2} + \left( {2l\cos \theta - x} \right)^{2}$$

$$x = 2l\cos \theta - 2\sqrt {l^{2} - \left( {l\sin \theta + y/6} \right)^{2} }$$

Define position a is the equilibrium state and initial force of the spring *F*_0_, then

$$F_{0} = \frac{\text{Mg}}{3\tan \theta }$$

When the mass *M* is moving downwards to the position *b*, the restoring force when the mass is at position b is

$$F = 3\left( {F_{0} + k_{l} x} \right)\tan \left( {\varphi + \theta } \right) - {\text{Mg}}$$

The force can be presented by *y*

$$F = 3\left( {\frac{Mg}{3\tan \theta } + k_{l} \left[ { - 2l\cos \theta + 2\sqrt {l^{2} - \left( {l\sin \theta - y/6} \right)^{2} } } \right]} \right)\frac{2l\sin \theta - y/3}{{2\sqrt {l^{2} - \left( {l\sin \theta - y/6} \right)^{2} } }} - Mg$$

Thus, the curve of nonlinear restoring force with different displacement y of the bio-inspired 3 layer leg suspension structure can be present in the following figure.

**Fig. 7:**
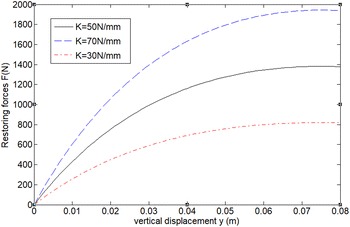
Nonlinear restoring force with different displacement y of the bio-inspired 3-layer leg suspension structure

From Fig. 7, it can be seen that (a) the equivalent stiffness of the X-shaped structure is nonlinear and the nonlinear stiffness is decreased with the compression of the structure increasing; (b) for the same structure, higher spring stiffness leads to higher loading capacity but the equivalent stiffness can all be close to zero after the structure is compressed to certain distance. As far as we know, this nonlinear stiffness property is unique in all existing spring system irrespectively of traditional springs or pneumatic springs, as most existing spring systems have obviously increased stiffness during compression. This beneficial stiffness offers excellent dynamic stiffness of the robot “legs” when the payload is increased.

### Dynamic analysis

To understand the dynamic stiffness of the X-structured suspension, the dynamic model is studied in this section. It is supposed that the loading mass is *M*, the rod length is *l* and at equilibrium the assembly angle *θ* denotes the angle between the rod and the horizontal line. The spring stiffness is denoted by *k*_*l*_. The basic parameter for the 3-layer bio-inspired leg suspension model is listed in the following Table 1.

**Table 1:** Parameter for the *n*-layer bio-inspired leg suspension model

Symbol	Structural parameters	Unit
*k*_1_	Linear stiffness	N m^−1^
*M*	Loading mass	Kg
*l*	Length of connecting rod	m
*θ*	Deigned working angle	rad

Moreover, the absolute motion of the loading mass *M* is represented by *y*, the bottom excitation *z*, the rotation angle of each connecting rod *φ*, and the horizontal motion of the joints at each layer *x*. The positive direction of *y* is upward in the modeling. All motion variables are listed in Table 2.

**Table 2:** Motion variable for the *n*-layer bio-inspired leg suspension model

Symbol	Structural parameters	Unit
*y*	Absolute motion of loading mass	m
*z*	Bottom excitation	m
$\hat{y}$	Relative motion of loading mass and bottom	m
*φ*	Rotational motion	rad
*x*	Horizontal translational motion	m
*z*_0_	Amplitude of bottom excitation z	m
*ω*_0_	Frequency of bottom excitation z	rad s^−1^
*ϕ*	Phase of relative motion *ŷ*	
*a*	Amplitude of relative motion *ŷ*	
*T*_*d*_	Displacement transmissibility	

The detailed modeling process of the n-layer symmetric X-shaped structure can be referred to [12, 14]. Here, it is applied to the 3-layer structure in Fig. 6. For convenience in discussion and for understanding dominant dynamic response of the system, the mass of the connecting rods is not considered. The absolute motion of the isolation object *y* is the generalized coordinate. The kinetic energy can be written as

$$T = \frac{1}{2}M\dot{y}^{2}$$

The potential energy is

$$V = \frac{1}{2}k_{l} x^{2}$$

The Lagrange principle is

$$\frac{\text{d}}{{{\text{d}}t}}\left( {\frac{\partial L}{{\partial \dot{y}}}} \right) - \frac{\partial L}{\partial y} = - D$$

In which *L* = *T* − *V*, and *D* is the overall damping effect which is considered as a linear component as *D* = $c\left( {\dot{y} - \dot{z}} \right)$. From Fig. 6, the relative motion between the mass M and the bottom is *ŷ* = *y*–*z* where *y* is the absolute motion of the mass *M* and *z* the bottom excitation, the geometrical relation of variables *φ*, *x* and *ŷ* can be obtained as

$$\tan \left( {\varphi + \theta } \right) = \frac{{2l\sin \theta + \hat{y}/3}}{2l\cos \theta - x}$$

$$\left( {2l} \right)^{2} = \left( {2l\sin + \hat{y}/3} \right)^{2} + \left( {2l\cos \theta - x} \right)^{2}$$

The motion $\varphi$ and *x* can be expressed as

$$\varphi = \arctan \left[ {\frac{{2l\sin \theta + \hat{y}/3}}{2l\cos \theta - x}} \right] - \theta$$

$$x = 2l\cos \theta - 2\sqrt {l^{2} - \left( {l\sin \theta + \hat{y}/6} \right)^{2} }$$

By substituting kinetic energy, potential energy, into the Lagrange principle, the dynamic equation of the bio-inspired leg suspension can be obtained as

$$M\ddot{y} + \left( {k_{l} x} \right)\frac{{{\text{d}}x}}{{{\text{d}}\hat{y}}}\frac{{{\text{d}}\hat{y}}}{{{\text{d}}y}} = - c\left( {\dot{y} - \dot{z}} \right)$$

In which $\dot{\varphi } = \frac{{{\text{d}}\varphi }}{{{\text{d}}\hat{y}}}\frac{{{\text{d}}\hat{y}}}{{{\text{d}}t}}$ and $\dot{x} = \frac{{{\text{d}}x}}{{{\text{d}}\hat{y}}}\frac{{{\text{d}}\hat{y}}}{{{\text{d}}t}}$ due to the geometrical relations. After some manipulation, the dynamic equation can be rewritten as

$$M\ddot{\hat{y}} + c\dot{\hat{y}} + \beta_{1} \hat{y} + \beta_{2} \hat{y}^{2} + \beta_{3} \hat{y}^{3} = - M\ddot{z}$$

where the base excitation z = z_0_ cosω_0_t, and

$$\beta_{1} = \frac{{k_{l} \tan^{2} \theta }}{{n^{2} }}$$

$$\beta_{2} = \frac{{3k_{l} \,\text{sec}^{4} \theta \sin \theta }}{{4n^{3} l}}$$

$$\beta_{3} = \frac{{k_{n} }}{{n^{4} }} - \frac{{4{\left(k_{l} + 2k_{n} l^{2} \cos 2\theta\right)} \,\text{sec}^{3} \theta - 5k_{l} \,\text{sec}^{5} \theta }}{{8n^{4} l^{2} \cos \theta }}$$

**Fig. 8:**
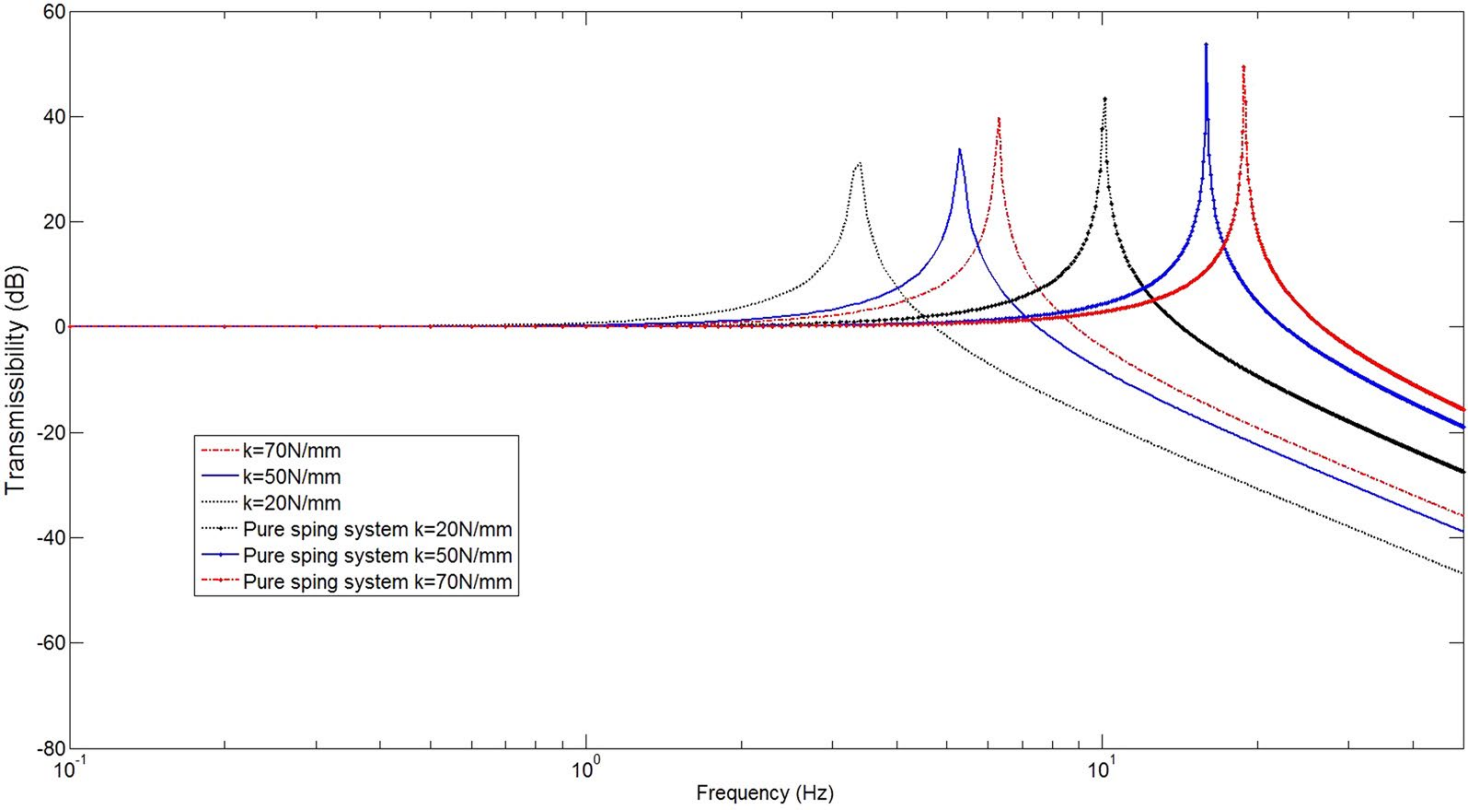
Transmissibility curves with the theoretical analysis

The transmissibility curves are shown with different stiffness in Fig. 8. It can be seen that with smaller *k* a smaller resonant frequency can be obtained indicating a better vibration isolation performance. Compared with the system of the same stiffness *k*_l_ but without using the X-shaped structure, the resonant frequency is significantly reduced, indicating a much improved vibration isolation performance (Table 3). This is very good for robot body stability moving on rough grounds. Moreover, the structure can also be tuned with different assembly angle such that a smaller dynamic stiffness can be obtained. This is discussed in the following section.

**Table 3:** Dimensionless parameters of the bio-inspired leg suspension model

Dimensionless parameters	Value
*ω*_1_	$\sqrt {k_{l} /M}$
*t’*	*ω*_1_
*ω*_2_	$\sqrt {\beta_{1} /M}$
*γ*_1_	$\beta_{2} /M$
*γ*_2_	$\beta_{3} /M$
$\varOmega$	*ω*_0*/*_ *ω*_1_

### Equivalent stiffness and resonant frequency

The overall spring force of the X-structure is obviously a nonlinear function, and from the dynamic modeling, the equivalent linear and nonlinear stiffness coefficients *β*_1_ *, β*_2_ *, β*_3_ can be obtained as.

$$\beta_{1} = \frac{{k_{l} \tan^{2} \theta }}{9}$$

$$\beta_{2} = \frac{{3k_{l} \,\text{sec}^{4} \theta \sin \theta }}{108l}$$

$$\beta_{3} = \frac{{4k_{l} \,\text{sec}^{3} \theta - 5k_{l} \,\text{sec}^{5} \theta }}{{648l^{2} \cos \theta }}$$

The linear stiffness coefficient *β*_1_ is a dominant factor to represent the resonant frequency of the system. To have a better vibration isolation performance, this coefficient should as small as possible [18, 19]. The resonant frequency is (Hz)

$$f_{0} = \frac{1}{2\pi }\sqrt {\frac{{\beta_{1} }}{M}} = \frac{\tan \theta }{6\pi }\sqrt {\frac{{k_{l} }}{M}}$$

This equation reveals very clearly that the resonant frequency of the single bio-inspired leg suspension can be tuned to be very small with a smaller *k*_l_, a bigger payload M or a smaller assembly angle θ. When a higher payload is applied, the spring stiffness *k*_l_ could be required to increase. In such case, a smaller assembly angle θ can be used for a much smaller dynamic stiffness and thus a much softer single “leg” in moving while maintaining a higher loading capacity. This is a significant property that cannot be seen in all existing passive suspension systems.

### Loading capacity

The loading capacity is determined by the stiffness of the horizontal spring *k*_l_, assembly angle *θ*, preload force and strength of structure. Define *F*_0_ was the initial force of the spring and *θ*_2_ is the minimum working angle of the system, when the model is at equilibrium, $3F_{0} \tan \theta = {\text{Mg}}$. Then, from the assembly angle *θ*, compressed to the minimum working angle, the loading should be applied by $2lk_{l} \left( {\tan \theta - \tan \theta_{2} } \right)$, i.e., $F_{0} + 2lk_{l} \left( {\tan \theta - \tan \theta_{2} } \right) \ge \frac{\text{Mg}}{{3\tan \theta_{2} }}$.For the 3-layer bio-inspired leg suspension,

$${\text{Mg}}\left( {\frac{1}{3} - \frac{1}{{3\tan \theta_{2} }}} \right) \le 0.1k_{l} \left( {1 - \tan \theta_{2} } \right).$$

$$M \le \frac{{0.3k_{l} \left( {1 - \tan \theta_{2} } \right)}}{{\left( {1 - \cot \theta_{2} } \right)g}}$$

With the current design, the maximum loading of the single bio-inspired leg suspension can be 8 kg. Accordingly, the overall robot will have the loading capacity up to 80 kg.

## ADAMS Model

**Fig. 9:**
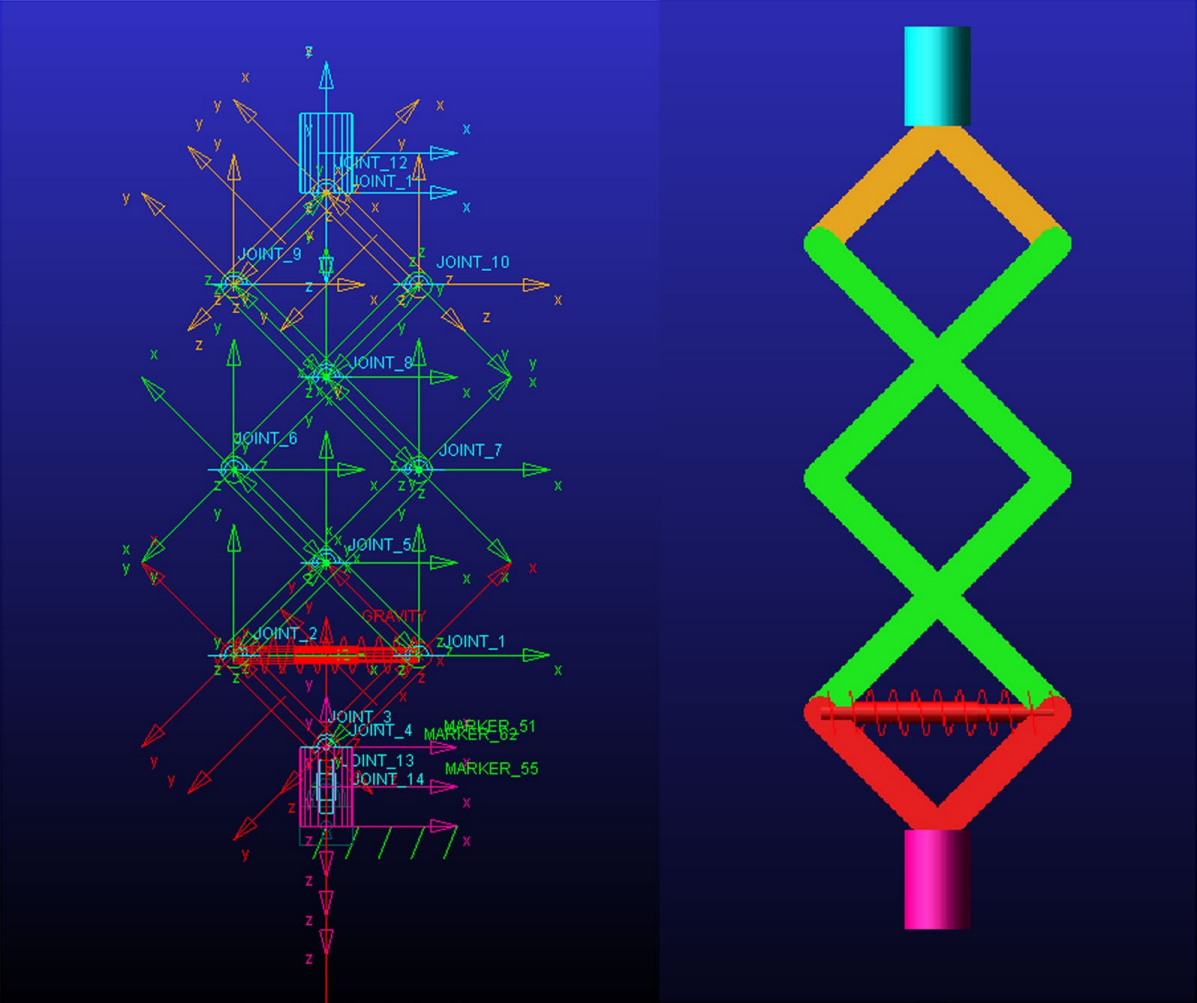
A 3-layer bio-inspired suspension in the ADAMS model

**Fig. 10:**
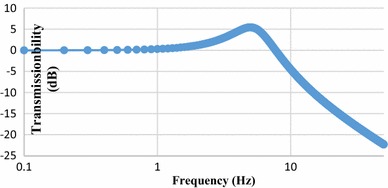
Transmissibility curves under the simulation environment in Adams

The model built in ADAMS is as shown in Fig. 9 and Fig. 10 which is used to verify the theoretical analysis above.

With the loading mass *M* = 5 kg, spring stiffness *k*_l_ = 50000 N/m, rod length *l* = 0.05 m and working angle at 45°. The transmissibility curve can be achieved by the simulation in Adams which indicates a resonant frequency 0.6 Hz (Fig. 10).

As the theory analysis above, the resonant frequency of the system is

$$f_{0} = \frac{1}{2\pi }\sqrt {\frac{{\beta_{1} }}{M}} = \frac{\tan \theta }{6\pi }\sqrt {\frac{{k_{l} }}{M}}$$

Thus,

$$f_{0} = 5.3\,{\text{Hz}}$$

The result obtained by the simulation 5.4 Hz matched the theory analysis 5.3 Hz.

## Experiment study

In this section, the vibration isolation performance of the 3-layer passive bio-inspired suspension is verified. Moreover, the overall robot will be tested by the resonant frequency and obstacle passing ability.

### Single 3-Layer bio-inspired suspension

**Fig. 11:**
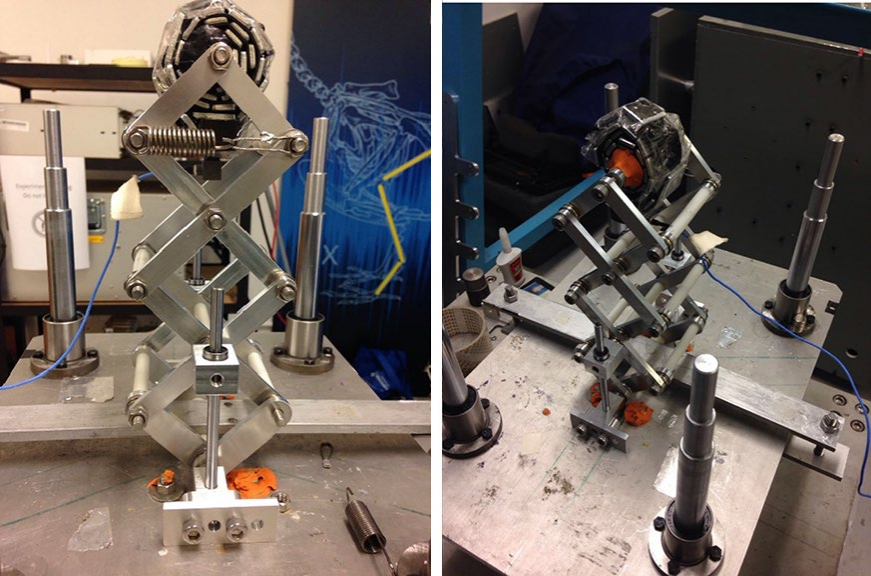
Prototype of the single 3-layer bio-inspired leg suspension

For the single 3-Layer bio-inspired suspension, an upper mass *M* = 0.5 kg is attached to the top of the bio-inspired leg as shown in Fig. 11, and the horizontal linear spring’s stiffness is *k*_l_ = 4800 N/m. A random vibration signal is generated with a vibration shaker vertically at the bottom platform. The experiments will be repeated several times to guarantee the result.

**Fig. 12:**
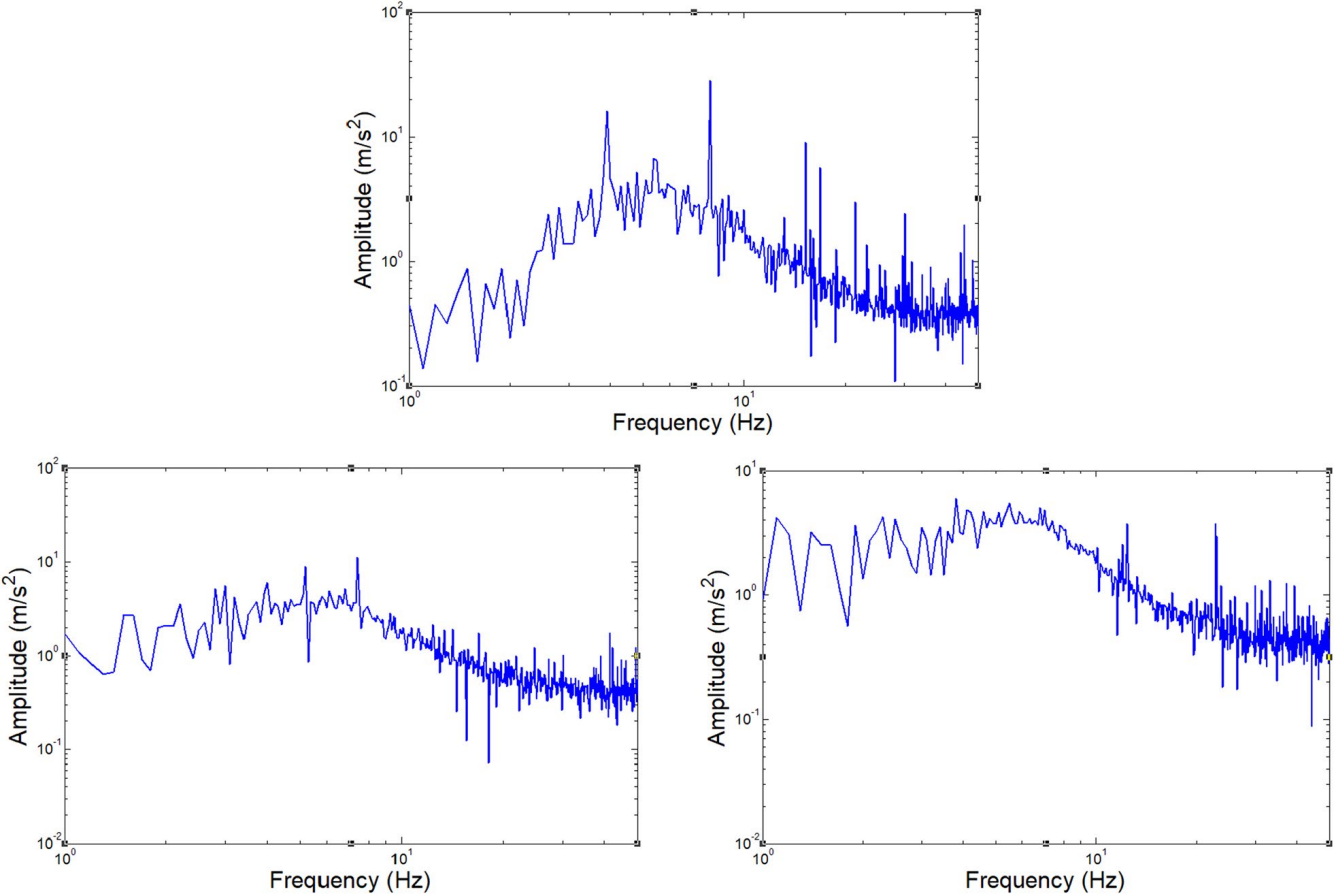
Vibration transmissibility of the upper mass in the single 3-layer bio-inspired leg suspension

The acceleration of the upper mass can then be measured with transmissibility shown in Fig. 12 (3 repeated times), which show the resonant frequency of the system is about 5.5 Hz. In theoretical analysis, the resonant frequency can be represented by

$$f_{0} = \frac{1}{2\pi }\sqrt {\frac{{\beta_{1} }}{M}} = \frac{\tan \theta }{6\pi }\sqrt {\frac{{k_{l} }}{M}}$$

where *k*_l_ = 4800 N/m, *M* = 0.5 kg, θ = 45°, then *f*_0_ = 5.2 Hz, which means the theory analysis can match the experiment results.

Note that for a pure spring-mass system, the resonant frequency would be around 15.6 Hz. This greatly validates that the bio-inspired leg can support the same payload but have much smaller dynamic stiffness and thus much better vibration isolation performance. This property will be greatly helpful for stability and comfort of the upper platform of the robot during moving on rough ground.

### Overall robot with 2-layer bio-inspired suspension

**Fig. 13:**
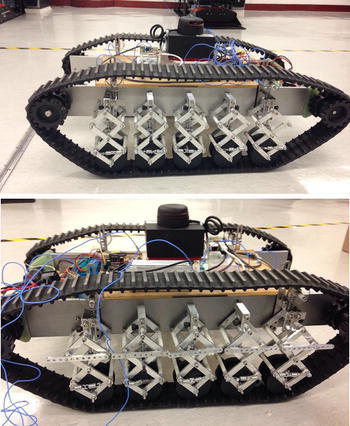
Overall robot with 2-layer bio-inspired leg suspension

In this section, comparative experiments are done to study the vibration response to road bump with a developed 2-layer bio-inspired suspension. For comparisons, the passive bio-inspired suspension can be activated or locked as shown in Fig. 13. The robot will be controlled to passive an obstacle about 2–3 cm high on a small laboratory ground. In such case, any small obstacle could be observed clearly on the response of the acceleration on the robot body. Two cases will be considered. The one is that the robot is to pass the obstacle with only one side of the wheels, and the other is to pass the obstacle with both sides of the wheels.

The overall equivalent load of the whole system *M* = 10 kg, the speed *v* = 0.9 m/s, the stiffness of any single horizontal spring is 3500 N/m in each single “leg.” In such case, the resonant frequency in the vertical direction of the robot would be around 4.7 Hz based on theoretical calculation.

#### Pass obstacles with two tracks

In this section, the robot runs over the obstacle with both of its tracks. The overall payload of the ten-leg suspension is *M*_*l*_ = 10 kg, the robot speed is *v* = 0.9 m/s, and the stiffness of the horizontal spring was 3500 N/m. The height of the obstacle is about 3 cm. The time when the robot touches the obstacle on the ground is between 4 and 5 s.

**Fig. 14:**
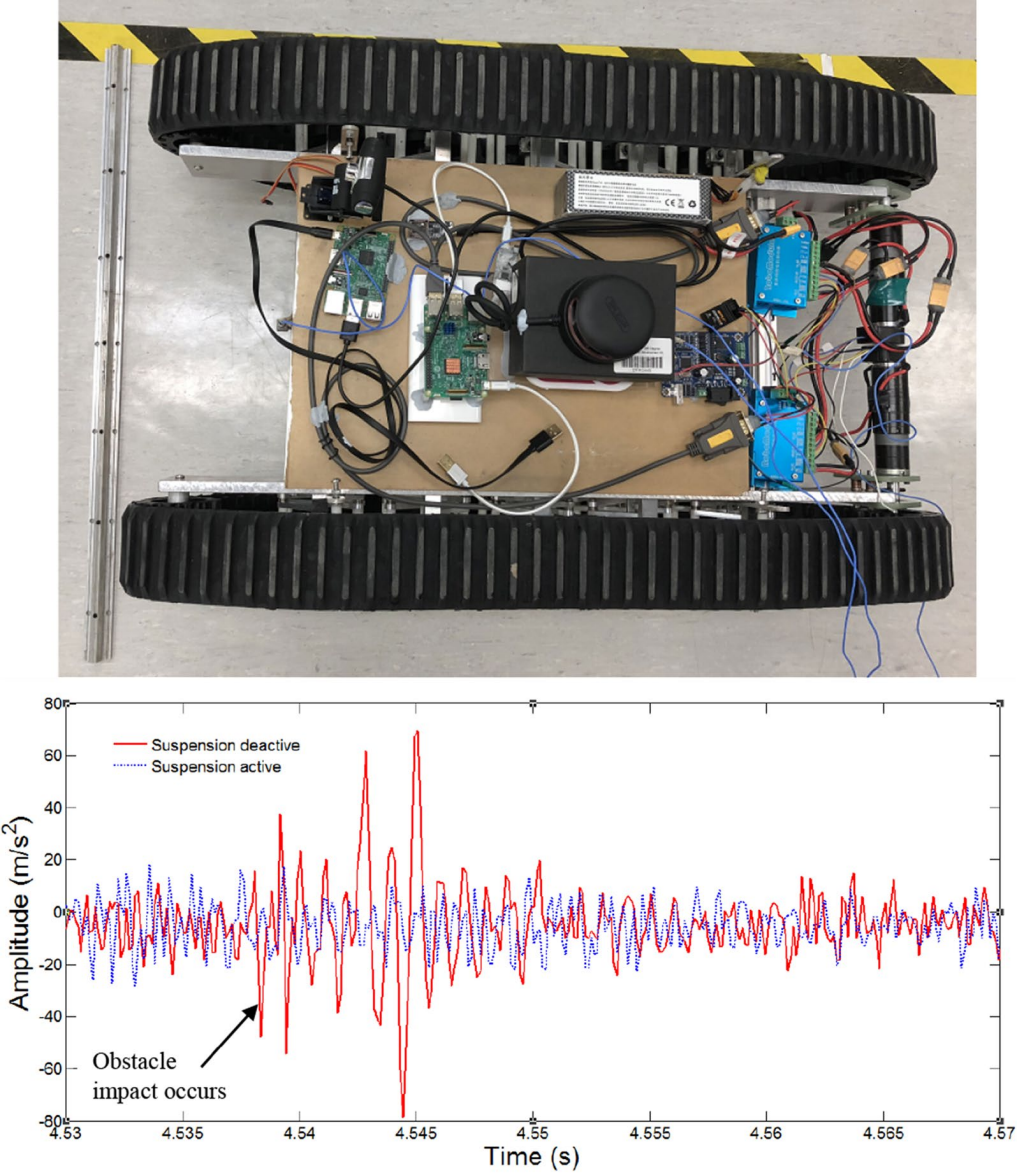
Pass one bump obstacle with two tracks and the vibration response of the upper platform of the robot with 2-layer bio-inspired suspension

**Fig. 15:**
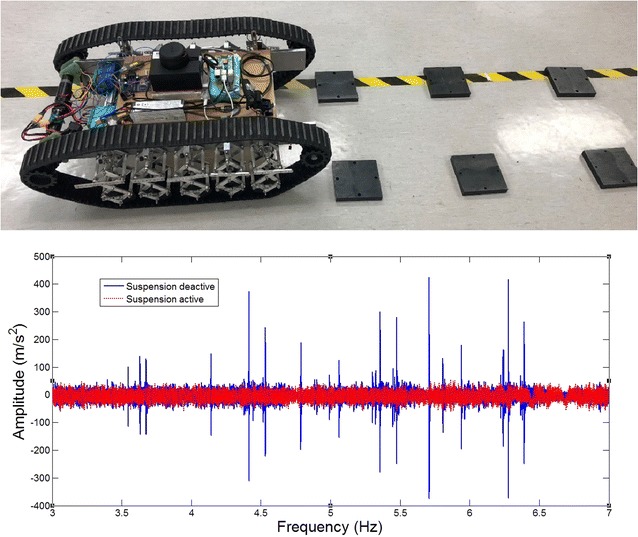
Passing multi-obstacles with both tracks and the vibration response

The acceleration response of the upper platform with the suspension switched on can be attained by the three sensors. Compared with the data with the suspension locked-off, it can be clearly seen that the robot is much more stable with the bio-inspired suspension system (Fig. 14). Similar results can also be seen when the robot passes multi-obstacles with both tracks. Without the bio-inspired suspension, the robot has very strong impulse response at the upper platform (Fig. 15).

When the bio-inspired suspension is used, the robot passes the obstacle without any obvious response, while the robot has obvious vibration without the suspension.

#### Pass obstacles with one track

**Fig. 16:**
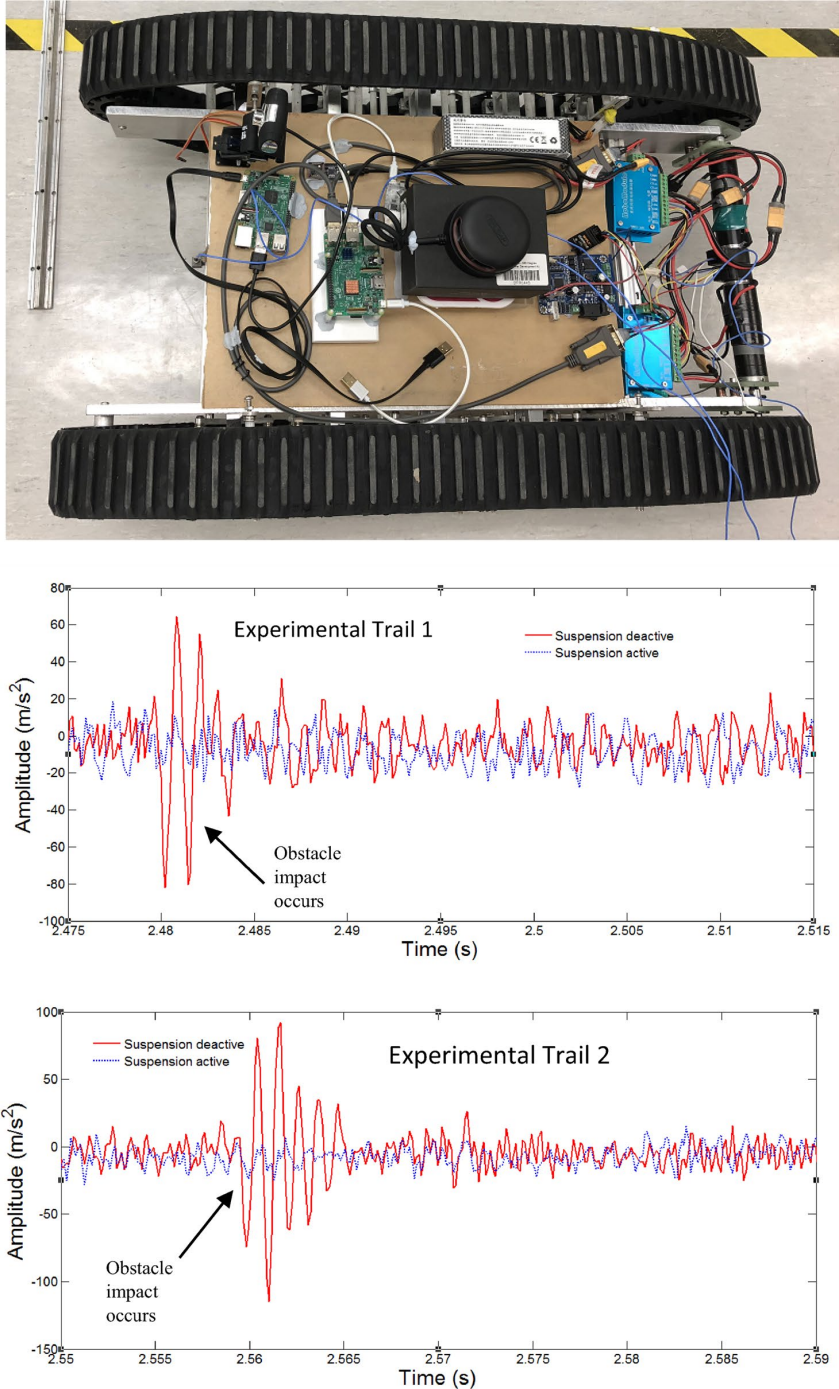
Pass obstacles with only one track and the vibration response of the upper platform of the robot with 2-layer bio-inspired suspension

In this section, the robot runs over the obstacle with only one track passing on. All parameters are the same as before (Fig. 16).

**Fig. 17:**
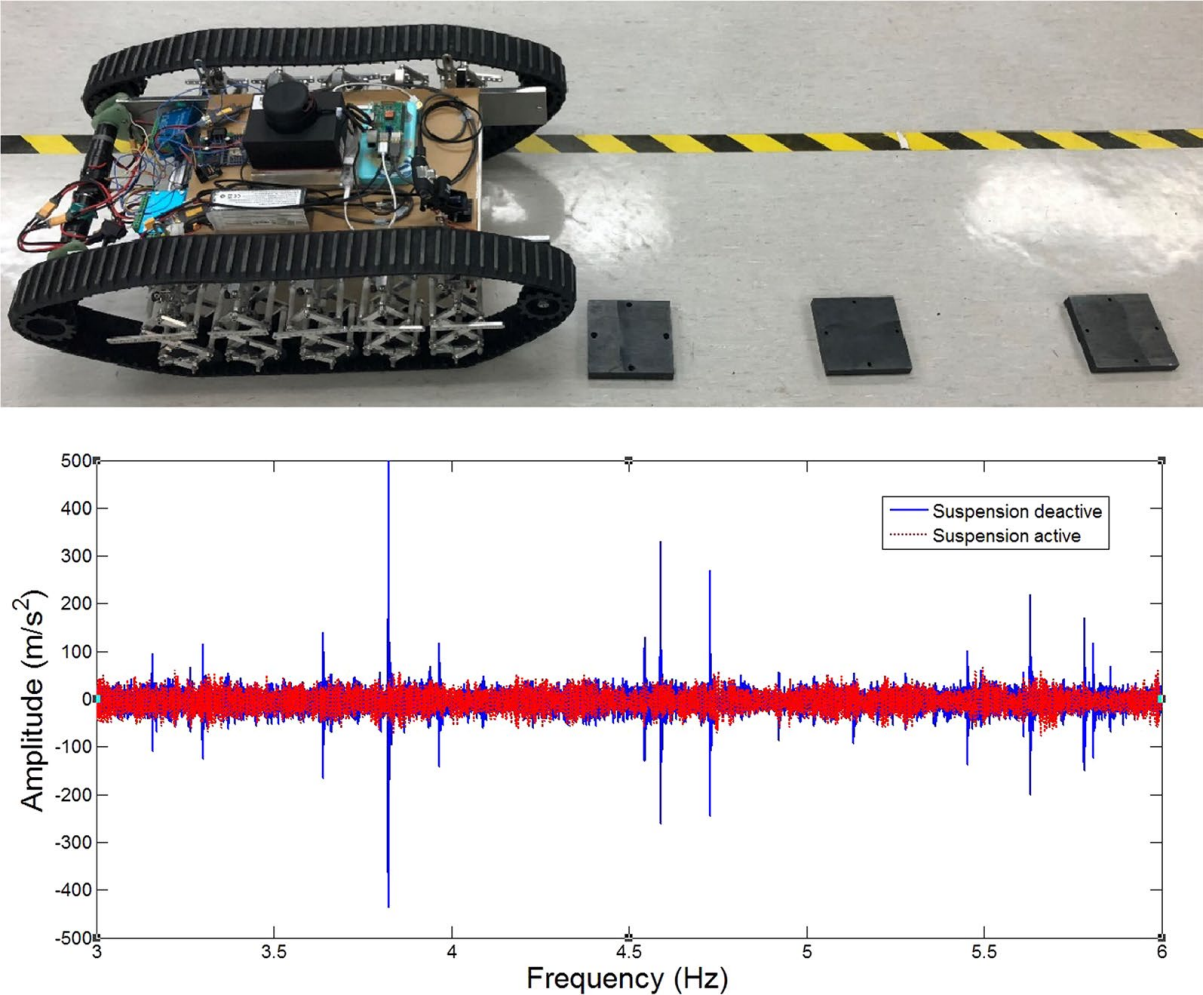
Passing multi-obstacles with only one track and the vibration response

The results are shown in Fig. 16 with two trials, both indicating very good performance during passing the bump obstacle. The robot with the passive bio-inspired suspension has almost no response to the bump when passing on but the robot responded strongly to the bump without the suspension design. Similar results can also be seen in Fig. 17 where the robot passes multi-obstacles with only one track. Very clear shock response can be seen without the bio-inspired suspension.

These experiments demonstrate clearly that the bio-inspired passive suspension is very helpful for vibration control of the upper platform of the robot.

## Conclusions and discussion

In this study, the track-based mobile robot with a novel passive bio-inspired leg-like suspension is designed and demonstrated both with theoretical analysis and experiments. It is shown that

1. The bio-inspired X-shaped or leg-like structure has very good static loading capacity and simultaneously very excellent quasi-zero dynamic stiffness properties, which are very good for vibration control of the robot body in a pure passive manner;
2. The bio-inspired structure is successfully applied to the passive suspension design of the track-based mobile robot such that the robot can maintain high stability, trafficability, comfort and loading capacity simultaneously, with only a passive structure design.

Due to the advantages above, the track-based robot with this novel passive bio-inspired suspension can be extensively used as a universal intelligent platform for various engineering tasks on rough ground.

It should be noted that the bio-inspired suspension is very good for vibration control but also creates other issues in accurate position control of the robot. When the robot goes through an obstacle, the belt would be fastened locally around or across the obstacle, the corresponding motor encoder can record the driving wheel rotation but actually the robot may not pass the recorded distance. Therefore, advanced robust tracking or position control based on multi-sensor information would be developed for these uncertainties in the further study.
